# Epidemiologic, clinical, diagnostic and therapeutic aspects of visceral leishmaniasis in renal transplant recipients: experience from thirty cases

**DOI:** 10.1186/s12879-015-0852-9

**Published:** 2015-02-25

**Authors:** Avelar Alves de Silva, Álvaro Pacheco E Silva Filho, Ricardio de Castro Cinta Sesso, Ronaldo de Matos Esmeraldo, Cláudia Maria Costa de Oliveira, Paula Frassinetti Castelo Branco Camurca Fernandes, Rodrigo Alves de Oliveira, Leila Silveira Veira de Silva, Valencio Pereira de Carvalho, Carlos Henrique Nery Costa, Jesusmar Ximenes Andrade, Diana Marisa Barros da Silva, Roosevelt Valente Chaves

**Affiliations:** General Clinic Department, Federal University of Piauí, Piauí, Brazil; Renal Transplant Unit, Hospital Alianca Casamater, Piauí, Brazil; Discipline of Nephrology, Federal University of São Paulo, São Paulo, Brazil; Renal Transplant Unit, Hospital Israelita Albert Einstein, Sao Paulo, Brazil; Renal Transplant Unit of the General Hospital of Fortaleza, Ceará, Brazil; Cantídio Walter University Hospital, Renal Transplant Unit, University Federal do Ceará, Ceará, Brazil; Division of Nephrology, Renal Transplant Service, Dr. Joaquim Bezerra Unit, School of Medicine, University of Crato, Ceará, Brazil; Infectious Diseases Hospital Dr. Natan Portela, Federal University of Piauí, Piauí, Brazil; Department of Accounting and Administration, Federal University of Piauí, Piauí, Brazil

**Keywords:** Visceral leishmaniasis, Renal transplant recipients, Infection after transplant

## Abstract

**Background:**

Visceral leishmaniasis is a disease caused by the protozoan *Leishmania* sp*.* and is transmitted by *Lutzomyia longipalpis* (sand fly). In renal transplant recipients, visceral leishmaniasis causes severe damage to the liver, spleen, and hematopoietic system, as well as poor outcomes for patients with transplanted kidneys. This study describes the largest series of cases of visceral leishmaniasis in renal transplant recipients, providing important information about the diagnostic routines and therapeutic strategies in this patient population.

**Methods:**

A retrospective, descriptive study was performed to analyze the distribution and evaluate the extent of the epidemiologic, clinical, diagnostic and therapeutic aspects of 30 renal transplant recipients from endemic regions who presented with visceral leishmaniasis in the post-transplantation period.

**Results:**

In this study, visceral leishmaniasis was more frequent in men (80%). The mean age of presentation was 40 ± 10.5 years. The majority of patients worked in urban areas (66.7%), cohabitated with domestic animals (90%), and were from low-income households. In 73.3% of cases, diagnosis was made by direct isolation of *Leishmania* forms. Patients were treated with liposomal amphotericin, resulting in a high degree of disease remission (80%).

**Conclusions:**

This study describes the largest series of visceral leishmaniasis in renal transplant recipients and expands clinical-epidemiological knowledge for transplantation teams to perform adequate disease management for this specific patient population.

## Background

Visceral leishmaniasis (VL) is an opportunistic disease caused by a protozoan of the genus *Leishmania* [1,2]. The disease is endemic to all continents; specialists consider it a neglected disease, as there are more than 5 million cases annually and the disease has a high incidence among the low socioeconomic, immunosuppressed, and malnourished population [2,3]. In Brazil, the incidence of VL is 2 cases per 100,000 inhabitants and in the states of Piauí and Maranhão, located in the Northeast of Brazil, incidence rises to 4 cases per 100,000 inhabitants [4]. Over the past decade, the mean annual cases in Brazil were 3,156, with 66% of all cases occurring in the Northeast and 48% in the states of Piauí and Ceará [4]. Of note, less than 100 cases of VL after kidney transplantation have been reported previously [5]. Brazil performs 8,000 kidney transplants per year; however, VL is a rare endemic disease and there is no national data of its incidence and prevalence in renal transplant recipients. Several studies have shown that in endemic areas transplant recipients might contract leishmania during transplantation, which might remain asymptomatic for a long period or for life. One cause of this is that serology for leishmaniasis is not yet a part of the pre-transplant evaluation protocol in many transplant centers [6,7]. VL is more common in children (54.4%) and in males (60%), and it is common in areas with a dry climate, an annual rainfall less than 800 mm, and a physiographic environment composed of valleys and mountains. However, the VL incidence has increased in urban areas, especially in the peripheries of large urban centers [8]. In recent years, kidney transplantation, particularly renal replacement therapy, has been initiated in poor regions and is associated with the standardization of surgical techniques and the ease of access to immunosuppressors [9-11]. Thus, problems due to acute rejection and surgical complications have decreased in these regions. Meanwhile, endemic and opportunistic infections such as VL have become the major preoccupation of transplantation teams, because these infections are directly associated with both graft dysfunction and the survival of renal transplant recipients [12].

There are currently no clinical protocols for the diagnosis or treatment of VL-infected renal transplant recipients, and few studies have focused on the epidemiology and risk factors associated with the disease in this specific patient population [12,13]. This study of 30 cases constitutes the largest series of this patient population reported and aims to highlight the epidemiologic, clinical, diagnostic and therapeutic aspects of VL in renal transplant recipients, as well as contribute to the establishment of better practices in the clinical management of these patients, with the possibility of improving patient survival and reducing graft rejection.

## Methods

### Study sample and location

Thirty kidney recipients were studied between January 1989 and January 2013. Patients resided and were followed-up postoperatively in transplant centers in regions where VL is endemic. The study location was the states of Piauí and Ceará, which are located in the Southern Hemisphere in Northeastern Brazil [14].

### Study design

This was a descriptive, retrospective study showing the relative distribution of renal transplant recipients with post-transplant VL, focusing on epidemiologic, clinical, diagnostic and therapeutic aspects.

### Patient inclusion and exclusion criteria

All confirmed VL cases who met the inclusion criteria and agreed to participate were included. Two patients did not agree to participate in the study and were therefore excluded. Deceased patients were included or excluded following telephone contact and/or a domestic visit to direct family members or spouses, which included an invitation, explanation of the methodology and importance of the research, and agreement. There were 10 deceased patients at the time of data collection; only two were excluded, as their family members could not be located following a change in address. The study was initiated after informed consent of patients and family, and approval by the research ethics committee of the Hospital Geral de Fortaleza (HGF) and Casamater hospitals as well as the federal universities of Piauí and Ceará.

### Case definition

Patients (*n* = 30) had chronic renal disease and were undergoing dialysis or conservative treatment. Kidney transplantation occurred between January 1989 and January 2013, and VL was detected post-transplantation. The patients were transplanted in the kidney transplant units of the Hospital Geral de Fortaleza (HGF), Hospital Universitário Walter Cantídio (HUWC), Hospital Universitário de Barbalha of the Universidade Federal do Ceará, and Hospital Aliança Casamater, which are all located in the states of Ceará and Piauí in Northeastern Brazil.

### VL treatment

VL patients were treated with liposomal amphotericin at 4 mg/kg**/**day for 10 days, amphotericin B up to the maximum dose of 1 g, or *N*-methylglucamine at 30 mg/kg**/**day for 20 days [15,16].

### Clinical remission

VL patients with no symptoms, signs, or laboratorial alterations 6 months post-treatment were considered cured [17,18].

### Definition of relapse

Relapse was defined as the occurrence of clinical manifestations of VL and new laboratorial identification of *Leishmania* in cases previously treated and considered cured up to 6 months post-treatment [19].

### Graft dysfunction

Graft dysfunction was defined as an increase in serum creatinine 30% above the baseline values in biochemical analyses performed before VL treatment in the absence of other factors associated with acute kidney injury [20].

### Variables analyzed

The following variables were analyzed. General characteristics included age, sex, ethnicity, breeding of or cohabitation with domestic animals, and ornamental and/or fruit plant breeding grown indoors or outdoors. In this study, the cohabitation of patients with domestic animals was defined as patients raising and/or taking care of animals, as well as animal presence in the neighborhood of residence or workplace. Other variables included the existence or absence of paved roads, wastelands, regular waste collection, sewage, and electricity in the neighborhood of residence or at the workplace as well as rural or urban areas. Epidemiologic characteristics included education level, family income, type of housing, and awareness of human or canine VL. Clinical profile characteristics included the type of dialysis before transplant, post-transplantation blood transfusions, donor type, first transplantation or re-transplantation, immunosuppression protocol, bacterial and viral infections, graft rejection, common clinical manifestations in VL patients, VL diagnosis methods, drugs used for VL treatment, and response to therapeutics. Laboratory data included hematocrit, platelets, leukocytes, serum albumin, creatinine and urea. All laboratory tests were performed at the beginning (day 1), middle (day 5), and end (day 10) of VL treatment as well as 90 and 180 days post-VL treatment in cured patients. Response to therapeutics was classified as disease progression to complete remission, death, graft dysfunction or return to dialysis.

### Data collection

After approval from the hospitals’ respective ethics committees, data were collected through patient interviews and a structured questionnaire explained and proctored by the researchers. Detailed questions included demographics, routines and socioeconomic conditions. Interviews were performed in a private room at the ambulatory care unit and lasted about 30 minutes. If one or more family members were present, they were also allowed to participate in the interview. In cases of deceased patients, interviews were performed with the spouse or a direct family member during domestic visits. Recording procedures for clinical and laboratory variables, clinical evaluation, and VL treatment were reviewed and a new assessment of patients and grafts was performed at 6 months post-treatment.

### Statistical analysis

Statistical analyses were performed with the use of descriptive statistics including means, standard deviations, frequencies and percentages. To determine if data were distributed normally, tests of equal proportions among all variables (general, epidemiologic and clinical aspects as well as responses to therapy [21]) were performed using the *χ*^2^ test.

Friedman ANOVA was used to analyze laboratory data, because the normality of the data could not be validated. When a significant difference was found by Friedman ANOVA, the Wilcoxon test was used as a post hoc test for multiple comparisons to test the pairs of variables that differed significantly. In such cases, significance was tested after performing a Bonferroni correction in which *p*-values < 0.05 were divided by the number of comparisons made. The baseline values were compared with values at each time point, which led to an a priori significance of 0.0125 (0.05/4 comparisons) for all variables except albumin, which had an a priori significance of 0.0166 (0.05/3 comparisons). Differences were considered significant only when *p* ≤ 0.0125 (or 0.0166 in the case of albumin) by the Wilcoxon test [22,23]. The level of significance for all tests performed was *p <* 0.05.

## Results

### Patient characteristics

The general characteristics of the patients are shown in Table 1. The proportion of male patients with post-transplant VL was significantly greater than the proportion of female patients (80% vs. 20%, respectively; *p* = 0.001). Patient age ranged between 22 and 60 years with a mean ± SD of 40.07 ± 10.50 years. There was a statistically significant difference with respect to ethnicity (*p* = 0.002), with a low percentage of brown-skinned patients (3.3%). The majority (90%) of patients were undergoing hemodialysis and had undergone their first transplantation before VL. A total of 56.7% of transplants were from live donors, the majority of whom were male (66.7%). Parents accounted for 33.3% of donors. Diseases leading to chronic renal disease included arterial hypertension in 50% of cases or diabetes mellitus in 33.3% (*p* = 0.002).

**Table 1:** **General characteristics of 30 renal transplant recipients with post-transplant visceral leishmaniasis**

**Variable**	Mean (SD)*	***n*** **(%)**	***P-value*** **
Age (years)	40.07 (10.5)		*-*
Sex			
Male		24 (80.0)	*0.001*
Female		6 (20.0)	
Ethnicity			
White		13 (43.3)	*0.002*
Black		16 (53.3)	
Brown		1 (3.3)	
Kidney transplant			
First transplant		27 (90.0)	*0.001*
Re-transplant		3 (10.0)	
Donor type			
Alive		17 (56.7)	*0.585*
Dead		13 (43.3)	
Donor sex (all donors)			
Male		20 (66.7)	*0.099*
Female		10 (33.3)	
Degree of kinship of live donors			
Second degree		11 (36.7)	
Parents		10 (33.3)	0.043
Siblings		8 (26.7)	
Not related		1 (3.3)	
Disease causing CRD			
Diabetes mellitus		8 (26.7)	0.432
Arterial hypertension		3 (10.0)	
Unknown		5 (16.7)	
Chronic GN		5 (16.7)	
Other		9 (30.0)	
Dialysis before transplant			
Hemodialysis		27 (90.0)	0.001
Peritoneal dialysis		3 (10.0)	

CRD, chronic renal disease; GN, glomerular nephritis.

*Mean (SD), Mean (Standard Deviation).

**χ ^2^ test.

Categorical variables are reported as n (%).

### Epidemiologic characteristics

The epidemiologic characteristics of the patients are shown in Table 2. There were uniform distributions of areas of residence (*p* = 1.000) and workplace locations (*p* = 0.099). Only one patient had an advanced education (3.3%) (*p* = 0.003), and only one patient (3.3%) had a family income greater than US$ 601 (*p* = 0.002). There were no statistically significant differences with respect to VL (*p* = 0.200) between patients who lived with animals (63.3%) and those who did not (36.7%). There were significant differences between patients who lived with dogs, cats, chickens, and birds but not pigs, and those who did not (*p* = 0.005, *p* = 0.001, *p* = 0.016, and *p* = 0.005, respectively). The percentage of VL patients who had plants in or around the house was significantly different from the percentage of VL patients who did not (*p* = 0.000 and *p* = 0.000, respectively). Sanitation and hygienic conditions in housing were considered adequate in 90% of cases. The proportions of patients who were aware of VL disease in humans and the VL vector were not significantly different, whereas significantly more patients were aware of canine leishmaniasis (CL) (86.7%) compared with those who were not aware (*p* = 0.000).

**Table 2:** **Epidemiologic characteristics of 30 renal transplant recipients with post-transplantation visceral leishmaniasis**

**Variable**	***n*** **(%)**	***P-value*** *
Residency		
Rural area	15 (50.0)	*1.000*
Urban area	15 (50.0)	
Workplace		
Urban area	20 (66.7)	*0.099*
Rural area	10 (33.3)	
Transplantation center		
Ceará (HGF, HUWC, Barbalha)	21 (70.0)	*0.043*
Piauí (Casamater)	9 (30.0)	
Education level		
Basic	14 (46.7)	*0.003*
Intermediate	15 (50.0)	
Advanced	1 (3.3)	
Cohabitation with domestic animals		
Yes	19 (63.3)	*0.200*
No	11 (36.7)	
Cohabitation with dogs		
Yes	23 (76.7)	0.005
No	7 (23.3)	
Cohabitation with cats		
Yes	24 (80.0)	0.001
No	6 (20.0)	
Cohabitation with chickens		
Yes	22 (73.3)	0.016
No	8 (26.7)	
Cohabitation with birds		
Yes	23 (76.7)	0.005
No	7 (23.3)	
Cohabitation with pigs		
Yes	11 (37.9)	0.265
No	19 (63.3)	
Plant breeding at home		
Yes	27 (90.0)	0.000
No	3 (10.0)	
Plant breeding in surroundings		
Yes	27 (90.0)	0.000
No	3 (10.0)	
Awareness of human VL		
Yes	11 (37.9)	
No	19 (63.3)	0.200
Awareness of VL vector		
Yes	10 (33.3)	
No	20 (66.7)	0.099
Awareness of CL		
Yes	4 (13.3)	
No	26 (86.7)	0.000
Family income nonthly		
Low (<US$ 200)	15 (50.0)	0.003
Average (between US$ 201 and 600)	14 (46.7)	
High (>US$ 600)	1 (3.3)	

HGF, Hospital Geral de Fortaleza; HUWC, Hospital Universitário Walter Cantídio; VL, visceral leishmaniasis; CL, canine leishmaniasis*.*

*χ ^2^ test. Categorical variables are reported as n (%).

### Clinical characteristics

The clinical data of patients with post-transplantation VL are shown in Table 3. The mean number of blood transfusions before transplantation was 0.80 ± 0.66 and the mean time between transplantation and VL was 21.3 ± 16.14 months with a median of 28 months. The mean number of acute rejections was 0.93 ± 0.74. There was a significant difference between at least one blood serotype, with the proportion of serotype B (10%) apparently causing this difference. The majority of patients were Rh factor positive (73.3%; *p* = 0.001). The majority of renal transplant recipients with VL had less than three incompatibility mismatches with the donor (83.3%), whereas only 16.7% had a greater number of mismatches (*p* = 0.001). There was no significant difference between the percentages of patients who did and did not receive blood transfusions before transplantation (43.3% vs. 56.7%, respectively; *p* = 0.465). Regarding the use of immunosuppressors, prednisone was used in 100% of cases, whereas there were significant differences with respect to mycophenolate mofetil and azathioprine use (*p* = 0.001 and *p* = 0.001, respectively). In contrast, there were no significant differences in the percentages of patients using of tacrolimus or cyclosporine (*p* = 0.465 and *p* = 0.144, respectively). Significantly more patients underwent induction with monoclonal antibodies than those who underwent no induction (70% vs. 30% respectively; *p* = 0.028). There were no significant differences between the percentages of patients with and without acute rejection (40% vs. 60% respectively; *p* = 0.362). All cases studied were first-time VL patients. Regarding post-transplantation infections, cytomegalovirus infection occurred in 40% of patients (*p* = 0.362) and bacterial infections occurred in 36.6% of patients (*p* = 0.002).

**Table 3:** **Clinical characteristics of 30 renal transplant recipients with post-transplantation visceral leishmaniasis**

**Variable**	Mean (SD)*	***n*** **(%)**	***P-value*** **
Number of blood transfusions before RTx	0.80 (0.66)	-	*-*
Median time between RTx and VL (months)	28	-	*-*
Mean time between RTx and VL (months)	21.3 (16.15)	-	*-*
Acute rejection episode	0.93 (0.74)	-	*-*
Blood transfusion before RTx			
Yes		13 (43.3)	*0.465*
No		17 (56.7)	
Blood type of recipient			
Serotype O		14 (46.7)	*0.025*
Serotype A		13 (43.3)	
Serotype B		3 (10.0)	
Rh^∞^ factor of recipient			
Positive		22 (73.3)	*0.011*
Negative		8 (26.7)	
Incompatible mismatches, recipient–donor			
≤3 mismatches		25 (83.3)	*0.001*
>3 mismatches		5 (16.7)	
Use of prednisone			
Yes		30 (100.0)	*-*
No		0 (0.0)	
Use of azathioprine			
Yes		6 (20.0)	*0.001*
No		22 (80.0)	
Use of cyclosporine			
Yes		11 (36.7)	*0.200*
No		19 (63.3)	
Use of tacrolimus			
Yes		17 (56.7)	*0.565*
No		13 (43.3)	
Use of MMF^♯^			
Yes		27 (90.0)	*0.000*
No		3(10.0)	
Induction with monoclonal antibody			
Yes		21 (70.0)	*0.043*
No		9 (30.0)	
Acute rejection post-transplantation			
Yes		12 (40.0)	*0.362*
No		18 (60.0)	
Patients with VL before RTx			
Yes		0. (0.0)	
No		30 (100.0)	-
CMV^≠^ infection post-transplantation			
Yes		12 (40.0)	*0.362*
No		18 (60.0)	
Bacterial infection post-transplantation			
Yes		11 (36.6)	*0.002*
No		19 (73.4)	
Recipient with positive serology for other viruses			
No		20 (66.7)	*0.068*
Yes		10 (27.0)	

*RTx* renal transplantation, *VL* visceral leishmaniasis, *Rh* Rhesus factor, *MMF* mycophenolate mofetil, *CMV* cytomegalovirus, *Mean (SD)*, Mean (SD), Mean (Standard Deviation).

**χ ^2^ test. Categorical variables are reported as n (%).

### Symptoms and signs

The symptoms and signs detected in renal transplant recipients with post-transplantation VL are shown in Table 4. The percentages of patients differed significantly among each of the categories of symptoms and signs (*p* < 0.05), except for the existence of cavity fluids and edema. Even though 67.7% of patients had these signs, the difference was not statistically significant (*p* = 0.099).

**Table 4:** **Clinical manifestations in 30 renal transplant recipients with post-transplantation visceral leishmaniasis**

**Variable**	***n*** **(%)**	***P-value*** *
Fever		
Yes	21 (70.0)	*0.043*
No	9 (30.0)	
Weight loss		
Yes	30 (100.0)	*-*
No	0 (0.0)	
Skin lesions		
Yes	25 (83.3)	*0.000*
No	5 (16.7)	
Splenomegaly		
Yes	28 (93.3)	*0.000*
No	2 (6.7)	
Hepatomegaly		
Yes	21 (70.0)	*0.043*
No	9 (30.0)	
Active bleeding of the digestive tract mucosae		
Yes	7 (23.3)	*0.005*
No	23 (66.7)	
Jaundice		
Yes	6 (20.0)	*0.001*
No	22 (80.0)	
Pale skin and mucosae		
Yes	21 (70.0)	*0.043*
No	9 (30.0)	
Weakness and myalgia		
Yes	23 (76.7)	*0.005*
No	7 (23.3)	
Diarrhea		
Yes	26 (86.7)	*0.000*
No	4 (13.3)	
Fluids in visceral cavities		
Yes	20 (66.7)	
No	10 (33.3)	*0.099*

*χ ^2^ test.

Categorical variables are reported as n (%).

### Diagnosis

VL diagnosis was performed in 100% of patients (Table 5). *Leishmania* was directly isolated in 73.3% of cases, and indirectly detected (immunological test) in 26.7% of cases (*p* = 0.016). In addition, there were significant differences in the percentages of patients with respect to diagnostic method: myelogram (*p* = 0.011), antigen rK39 isolation (*p* = 0.000), polymerase chain reaction (PCR) (*p* = 0.000), and spleen biopsy (*p* = 0.000). Significantly more patients were treated with liposomal amphotericin (93.3%) than amphotericin B (6.7%; *p* = 0.000). In addition, most patients did not receive *N*-methylglucamine (23.3% vs. 76.7%, respectively; *p* = 0.005). After treatment, 26.7% of patients experienced VL relapse (*p* = 0.016). Furthermore, there were significant differences in the percentages of patients with respect to full cure (*p* = 0.001), death (*p* = 0.000), and achievement of cure with graft dysfunction (*p* = 0.016). No significant differences were observed for cure with graft loss (*p* = 0.099) or cure with return to dialysis (*p* = 0.099).

**Table 5:** **Diagnosis, treatment, and response to therapeutics in 30 renal transplant recipients with post-transplantation visceral leishmaniasis**

**Variable**	***n*** **(%)**	***P-value*** *
VL diagnosis		
Direct identification of the parasite	22 (73.3)	*0.016*
Indirect method (immunological)	8 (26.7)	
Myelogram		
Yes	19 (63.3)	*0.011*
No	11 (26.7)	
Antigen rk39 isolation		
Yes	5 (16.7)	*0.000*
No	25 (83.3)	
PCR		
Yes	1 (3.3)	*0.000*
No	29 (96.7)	
Spleen biopsy		
Yes	2 (6.7)	*0.000*
No	28 (23.3)	
Use of amphotericin		
Liposomal amphotericin B	28 (93.3)	*0.000*
Amphotericin B	2 (6.7)	
Use of *N*-methylglucamine		
Yes	7 (23.3)	*0.005*
No	21 (66.7)	
VL relapse		
Yes	8 (26.7)	*0.016*
No	22 (73.3)	
Patients with VL remission		
Yes	24 (80.0)	*0.001*
No	6 (20.0)	
Deceased patients		
Yes	5 (16.7)	*0.000*
No	25 (83.3)	
Patients with VL remission and graft loss		
Yes	10 (33.3)	*0.099*
No	20 (66.7)	
Patients with VL remission and graft dysfunction		
Yes	22 (73.3)	*0.016*
No	8 (26.7)	
Patients with VL remission and return to dialysis		
Yes	10 (33.3)	*0.099*
No	20 (66.7)	

*VL* visceral leishmaniasis,* rK39* antigen extracted from *Leishmania* sp., *PCR* polymerase chain reaction.

*χ ^2^ test.

Categorical variables are reported as n (%).

### Laboratory data

Laboratory data were analyzed for all patients (*n* = 30) at the initiation of the study (day 0) and at days 5 and 10 of treatment (Table 6). Additionally, the same examinations were performed 90 days and 180 days post-VL treatment for all cured cases (*n* = 24). Friedman ANOVA was performed to evaluate possible differences among the results of the hematological and biochemical parameters at different time points during VL treatment. Except for urea, which remained unchanged during treatment (*p* = 0.511), there were significant differences among all other parameters at different time points. Multiple pairwise comparisons between the baseline and subsequent time points by the Wilcoxon test with Bonferroni correction showed only creatinine increased significantly between baseline and day 5 (*p* = 0.007). Hematocrit differed significantly between baseline and day 10 (*p* = 0.000), day 90 (*p* = 0.000), and day 180 (*p* = 0.000), with an increase in red blood cells from day 10. Leukocytes and platelets also differed significantly between baseline and days 5, 10, 90, and 180. Even though albumin was significantly different by Friedman ANOVA, pairwise multiple comparisons between albumin levels at baseline and days 5, 90, and 180 did not reveal significant differences. This indicated albumin remained unchanged from the beginning of treatment until the time points studied (*p* = 0.962; *p* = 0.588, and *p* = 0.182, respectively).

**Table 6:** **Laboratory data of post-transplant visceral leishmaniasis patients in remission and up to 6 months post-transplantation**

**Variable**	Mean (SD)*	***P-value*** **
Creatinine (mg/dL)		*0.002*
1^st^ evaluation	1.81 (0.4)	
2^nd^ evaluation	2.21 (0.46)	
3^rd^ evaluation	1.9 (1.17)	
90 days post-treatment	1.77 (0.27)	
180 days post-treatment	1.80 (0.33)	
Urea (mg/dL)		*0.000*
1^st^ evaluation	105.13 (16.91)	
2^nd^ evaluation	97.80 (15.89)	
3^rd^ evaluation	65.13 (27.18)	
90 days post-treatment	63.40 (20.40)	
180 days post-treatment	52.07 (9.98)	
Hematocrit (%)		*0.000*
1^st^ evaluation	28.72 (4.18)	
2^nd^ evaluation	29.72 (3.65)	
3^rd^ evaluation	34.94 (3.50)	
90 days post-treatment	37.73 (2.57)	
180 days post-treatment	38.76 (1.72)	
Leukocytes (mm^3^/dL)		*0.000*
1^st^ evaluation	3.07 (1.05)	
2^nd^ evaluation	4.51 (3.27)	
3^rd^ evaluation	5.86 (2.71)	
90 days post-treatment	6.18 (1.41)	
180 days post-treatment	6.40 (0.95)	
Platelets (mm^3^/dL)		*0.000*
1^st^ evaluation	110.33 (88.18)	
2^nd^ evaluation	178.07 (183.17)	
3^rd^ evaluation	163.00 (72.02)	
90 days post-treatment	177.00 (18.13)	
180 days post-treatment	183.07 (11.88)	
Serum albumin (mg/dL)^†^		*0.009*
1^st^ evaluation	3.32 (0.76)	
2^nd^ evaluation	3.74 (0.37)	
90 days post-treatment	3.87 (0.34)	
180 days post-treatment	3.99 (0.27)	

*n* = 24 remission patients.

1^st^ evaluation: day 1 of treatment with amphotericin or glucamine.

2^nd^ evaluation: day 5 of treatment with amphotericin or day 10 with glucamine.

3^rd^ evaluation: day 10 of treatment with amphotericin or day 20 with glucamine.

^†^Serum albumin was not measured at intermediate time points.

*Mean and SD* (Mean (Standard Deviation) of leukocytes and platelets were divided by 1.000.

Categorical variables are reported as n (%).

**Determined by Friedman ANOVA, the Wilcoxon test or ANOVA factor repetition measurements.

## Discussion

Fewer than 100 cases of renal transplant recipients with VL have been reported [24,25]. This study investigated the largest number of VL-cases in renal transplant recipients to date. We hope this study of the epidemiologic, clinical, and diagnosis characteristics of VL will become a reference for transplantation teams to adequately follow-up renal transplant recipients with VL, as well as direct treatments, thus, reducing patient mortality and improving the preservation of graft function [11]. In this series, 80% of patients were men, who were more likely to be exposed to the mosquito vector of VL (*Lutzomyia longipalpis*) as a result of professional activities, greater body surface area, and the habit of remaining shirtless in the high-temperature environments characteristic of tropical regions [26,27]. Despite the lack of reports of an association between ethnicity and VL, the majority of patients in the present study were black. Only one previous study addressed the issue of the period between transplantation and VL diagnosis; however, there are no reports of an association between VL and the type or dose of immunosuppressors used. A recent case–control study of multivariate analysis of renal transplant recipients that coexisted with cats with or without VL, showed bacterial infections after transplant and inadequate socioeconomic conditions increased the risk for disease [11]. Other studies showed the mosquito vector had migrated from rural to urban areas [28,29]. The majority of studies in the literature report dogs are the main intermediary host in the life cycle of VL. However, cats are also important hosts of *Leishmania* [11]. VL is a neglected disease that is highly prevalent in the world’s poorest regions and is positively correlated with deficient hygiene and sanitation [30,31]. However, the present study shows that the majority of VL patients had access to purified water, electricity and regular waste collection. This suggests immunosuppression might be a determinant cause of VL in these patients, related to the use of immunosuppressors, hemodialysis, post-transplant infection, malnutrition and donor incompatibility, especially in cases of deceased donors [11,32,33]. Several studies failed to demonstrate a relationship between blood serotype and VL. The present study observed significant differences in the percentages of patients with respect to blood type and Rh factor, although there was no clinical explanation for this [34,35].

Previous studies reported difficulties in diagnosing VL in renal transplant recipients because of atypical signs and symptoms [36,37]. In the present study, clinical manifestations were typical, even in immunosuppressed patients, and disease occurred at a mean ± SD of 21.6 ± 16.15 months (range: 6–110 months) after transplantation with a median of 28 months. This contributed to the differential diagnosis of VL in cases of fever, a characteristic manifestation in all regions including non-endemic regions [38]. VL diagnosis was performed in 100% of cases, and myelogram was the main method used to identify *Leishmania* forms. In cases with negative results, indirect determination was performed by the identification of the rK39 antigen. Rapid diagnostic tests (RDTs) may improve the early detection of VL. Therefore, we evaluated the performance of a rK39-based RDT (Kalazar Detect™) for the detection of VL in an endemic, large urban area. Estimates for sensitivity and specificity were 72.4% and 99.6%, respectively [39]. Polymerase chain reaction using primers that amplify the conserved region of minicircle kDNA (DNA kinetoplast) is the most effective test for the diagnosis of LV, is minimally invasive as it uses blood samples from patients, and is superior to serology to detect cases of infection [7].

In the present study, liposomal amphotericin was the drug of choice for VL treatment, resulting in high levels of disease remission, low relapse and minimal numbers of deaths [39]. There is no consensus in the literature as to the amphotericin dose for the treatment of VL in renal transplant recipients. Previous studies used lower doses (20 mg divided in four doses and 1 mg/kg/day for 10 days) and shorter periods of time and observed a high cure rate of VL patients [40,41]. This study investigated cases of VL after renal transplantation diagnosed between 1989 and 2013. Cases with relapse (27,6%) were treated with N-methyl glucamine. The use of N-methyl glucamine was used in the first 7 cases of VL in kidney transplant because liposomal amphotericin was not used to treat VL [42,43]. Anti-leishmaniasis drugs are effective in treating disease but can cause significant side effects. Amphotericin B causes anaphylaxis, anemia, thrombocytopenia, nausea, vomiting, liver and renal failure, cardiac arrest and bronchospasm. Pentavalent antimony causes muscle and joint pain, diffuse abdominal pain, dyspnea, hepatic insufficiency, renal, pancreatic and electrocardiographic alterations [44,45].

The results of the current study showed an association between VL and post-transplantation bacterial and cytomegalovirus infections, which are explained by the reduced immunity of patients, who became more susceptible to opportunistic infectious organisms such as *Leishmania* [46]. As for immunosuppression, we found no significant difference among renal transplant recipients receiving cyclosporine or FK-506. These drugs have different chemical structures but similar mechanisms of action, blocking interleukins and activation of T and B lymphocytes, thus reducing patient immune responses [47,48].

Regarding laboratory analyses, the present study indicated that during systemic disease, VL seriously damages the kidneys, liver, spleen and hematopoietic system. Therefore, the early diagnosis and adequate treatment is critical for the significant improvement in patients.

## Conclusions

This study is the largest series of VL in renal transplant recipients, which focused on several aspects of the disease, including epidemiology, clinical profile, diagnosis, and treatment. The results advance our knowledge regarding VL in renal transplant recipients and may increase awareness of this emerging infection among transplantation teams. Standardized routines for the adequate follow-up of VL patients are recommended, taking into consideration the specificity of renal transplant recipients.
